# PRP INFILTRATION *VERSUS* CORTICOSTEROIDS: A PRELIMINARY RANDOMIZED CLINICAL TRIAL

**DOI:** 10.1590/1413-785220263404e295387

**Published:** 2026-07-17

**Authors:** William Zarza Santos, Roberta Sessa Stilhano Yamaguchi, Edilson Silva Machado, Rodrigo Góes Medéa de Mendonça, Alberto Gotfryd, Robert Meves

**Affiliations:** 1 Irmandade da Santa Casa de Misericordia de Sao Paulo, Departamento de Ortopedia e Traumatologia, Grupo de Afeccoes da Coluna Vertebral, Sao Paulo, SP, Brazil.

**Keywords:** Platelet-Rich Plasma, Low Back Pain, Injections, Adrenal Cortex Hormones, Plasma Rico em Plaquetas, Dor Lombar, Injeções, Corticosteroides

## Abstract

**Introduction:**

Chronic low back pain is one of the most prevalent health conditions, with significant social and economic impact. Its increasing incidence has driven the demand for new, effective therapeutic alternatives. This study compared the efficacy of platelet-rich plasma (PRP) and corticosteroid injections in the treatment of chronic low back pain of facet joint origin.

**Methods:**

This was a randomized clinical trial involving patients with chronic low back pain of facet joint origin, selected through a positive response to medial branch block. Epidemiological data and clinical outcomes were analyzed, including disability questionnaires (Roland-Morris Disability Questionnaire) and the visual analog scale (VAS) for pain. Assessments were conducted at baseline, one month, and three months post-procedure.

**Results:**

A total of 59 patients were included, with 32 in the corticosteroid group and 27 in the PRP group. No significant differences were found between the treatments in terms of disability scores or the Roland-Morris Questionnaire. However, a significant interaction between treatment and time was observed (p = 0.038). Regarding pain scores, no significant differences were found between groups at any of the assessed time points.

**Conclusion:**

The study found no evidence of superiority of platelet-rich plasma over corticosteroid injections in terms of pain reduction and functional outcomes for the treatment of lumbar facet syndrome. ***Level of evidence: I; Randomized clinical trial.***

## RESUMO

**Introdução:**

A dor lombar crônica é uma das condições de saúde mais prevalentes, com significativo impacto social e econômico. A sua crescente incidência tem gerado a necessidade de novas opções terapêuticas eficazes. Este estudo comparou a eficácia das infiltrações de plasma rico em plaquetas (PRP) e corticosteroides no tratamento da dor lombar crônica de origem facetária.

**Método:**

Trata-se de um ensaio clínico randomizado que avaliou pacientes com dor lombar crônica de origem facetária, selecionados por resposta positiva ao bloqueio do ramo medial, em relação aos efeitos dos tratamentos. Foram analisados dados epidemiológicos e desfechos clínicos, incluindo o Questionário de Incapacidade Roland-Morris e a escala visual analógica (EVA) de dor, com avaliações antes, 1 mês e 3 meses após o procedimento.

**Resultados:**

Foram incluídos 59 pacientes, 32 no grupo corticoide e 27 no grupo PRP. Não houve diferença significativa entre os tratamentos nos questionários de incapacidade e Roland Morris, embora tenha sido observada uma interação significativa entre tratamento e tempo (p=0,038). Na escala de dor, não houve diferença entre os tratamentos em nenhum dos tempos avaliados.

**Conclusão:**

O estudo evidenciou ausência de superioridade do Plasma Rico em Plaquetas em comparação ao corticoide na avaliação de dor e escores de função no tratamento da síndrome facetária lombar. ***Nível de evidência I; Estudo clínico randomizado.***

## INTRODUCTION

Low back pain is one of the most common health problems, with high prevalence and social cost^1^. Although we attempt to define low back pain based on lumbar structures (discogenic pain, facet joint pain, sacroiliac joint pain, myofascial pain), diagnostic investigations have a limited role. Both clinical tests show insufficient accuracy in this differentiation, as do imaging methods, which, although they reveal changes, often do not have clinical correlation with the type of pain^2^.

In light of the increasing cost, incidence, and prevalence of people with chronic low back pain, better treatment options have become a major point of discussion^3^. Treatment modalities such as corticosteroid injections have gained significant traction; however, there are limitations to this treatment regarding the frequency and duration of effect, as well as potential toxic properties to tendons and cartilage associated with these injections^4^.

In this context of high prevalence of low back pain, combined with the scarcity of effective treatment for the problem, the need for a therapeutic alternative with good clinical results arises, which presents lower morbidity and is supported by clinical trials^3^. This is precisely where biological therapies emerge as a promising alternative, with Platelet-Rich Plasma (PRP) and mesenchymal stem cells (MSCs) being the most prominent orthobiologics in the treatment of musculoskeletal pain.

The knowledge that platelets carry various growth factors and tissue regeneration factors led to the hypothesis that the application of a platelet concentrate could result in modulation of inflammation as well as a regenerative stimulus in an injured or degenerated tissue^5^. In the treatment of low back pain, the literature suggests benefits in the use of PRP^6^, however, what we still observe is a significant limitation of randomized clinical trials, especially related to facet joint pain, since most studies focus on intradiscal PRP injection aimed at discogenic low back pain^7^.

In addition to the scarcity of literature on randomized controlled clinical trials among patients with facet joint pain, it is also a fact that PRP is not yet a therapeutic method recommended by Brazilian medical authorities. Such factors increase the importance and relevance of the current work.

The objective of this work is to compare the efficacy of PRP with the standard treatment for facet joint pain: corticosteroid injection.

## MATERIALS AND METHODS

A double-blind randomized clinical trial was conducted, comparing the injection of platelet-rich plasma with the standard treatment (corticosteroid injection).

Patients over 18 years old with low back pain for at least 6 months and radiographs confirming degenerative changes in the lumbar spine were recruited for the study. Pain with a score of five or higher on the Visual Analog Scale (VAS) was also a criterion for recruitment in the study.

The following exclusion criteria were applied: pain radiating to the limbs (sciatica), previous lumbar spine surgery, radiographs showing spondylolisthesis, fractures, discitis, or tumors, pregnancy, history of drug abuse, allergy to lidocaine or corticosteroids.

To better select patients with facet joint pain, after patient recruitment, a test block of the medial branch of the facet joint was performed using 2% lidocaine, following the protocol of Rocha et al.^8^.

After ten minutes, the patient was asked to assess the intensity of pain using the VAS. If the pain improved by 50% or more, the block was considered positive and the patient was definitively included in the study. On the other hand, if after ten minutes the evaluated patient reported an improvement of less than 50% in pain on the VAS, the patient was excluded from the study.

After the patient selection, they were randomized into two blocks: Intervention with PRP or Intervention with corticosteroid, and they were kept blinded, not knowing which treatment they had received, just like the researcher, who administered the questionnaires.

For the preparation of PRP, peripheral venous blood was collected under aseptic technique in tubes containing anticoagulant of the type Citric Acid, Sodium Citrate, and Dextrose (ACD). The preparation followed the protocol with double centrifugation, as described by Machado et al.^9^.

**Figure 1:**
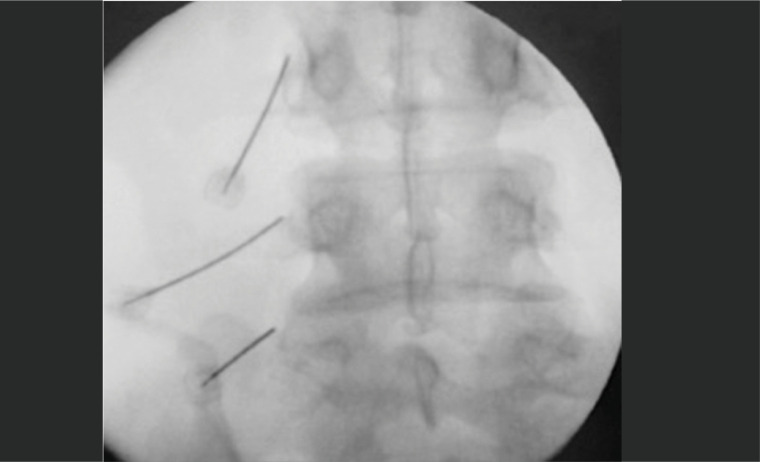
X-ray image of the procedure. X-ray image showing needle placement along the course of the medial branches of the dorsal rami at L3-L4, L4-L5, and L5-S1 on the left side.

The needles were positioned in the topography of the medial branches of the dorsal rami at L3-L4, L4-L5, and L5-S1 bilaterally, totaling six facet joints (Figure 1). Two mL of PRP or corticosteroid (Methylprednisolone Acetate) was applied to each articular facet. It is worth noting that, in both groups, one mL of 25% bupivacaine was also administered together with the injection in each articular facet.

Epidemiological data such as age, sex, BMI, physical activity, and presence of comorbidities were analyzed, in addition to clinical evaluations before the procedure, one month, and three months after the infiltration. During these periods, the Oswestry Disability Index (ODI)^10^ and Roland Morris (RM)^11^, as well as the Visual Analog Scale (VAS)^12^, were evaluated.

The interaction effects between the evaluation moment and treatment, the intervention effect, and the time effect were assessed through a repeated measures analysis of variance (ANOVA). In all statistical analyses, a significance level of 5% was adopted, meaning that results with a p-value less than 5% (p < 0.05) were considered statistically significant.

The research project was submitted for evaluation to the Ethics Committee on the Brazil Platform under CAAE 76715617.0.2001.5479 and approved by opinion 4.033.153.

It is emphasized that the refusal to accept the Informed Consent Form (ICF) or withdrawal of consent can occur at any time, without any consequences of any kind to the individual. Finally, all institutional rules established by resolutions 466/2012 and 510/2016 were strictly respected.

## RESULTS

### Total Sample

A total of 92 patients were evaluated, and of these, those who presented positive block (n = 60) were included. However, one of these patients did not attend the follow-up for evaluation, and thus was excluded from the protocol.

According to Table 1, it can be observed that the two groups were homogeneous concerning the group in all evaluated variables (p > 0.05). It can also be observed that, regardless of the group, most patients were female with an average age of 56 years, and did not engage in physical activity. Furthermore, most patients had comorbidities, with hypertension being the most common.

**Table 1:** Distribution of demographic and clinical data of patients evaluated in the study.

Variables	Groups		p-value
	PRP (n=27)	Corticosteroid (n=32)	
**Sex**			**0.200**
Male	10 (37.0%)	7 (21.9%)	
Female	17 (63.0%)	25 (78.1%)	
**Age, years**			**0.907**
Mean ± standard deviation	55.8 ± 10.7	56.1 ± 10.0	
**IMC, Kg/m^2^**			**0.733**
Mean ± standard deviation	28.5 ± 4.3	28.1 ± 4.0	
**Engages in physical activity**			**0.517**
No	17 (63.0%)	22 (71.0%)	
Yes	10 (37.0%)	9 (29.0%)	
**Comorbidities**			**0.192**
Absent	12 (44.4%)	9 (28.1%)	
Present	15 (55.6%)	23 (71.9%)	
Hypertension	11 (40.7%)	13 (40.6%)	0.993
Diabetes	7 (25.9%)	6 (18.8%)	0.508
Hypercholesterolemia	7 (25.9%)	5 (15.6%)	0.327
Hypothyroidism	1 (3.7%)	0 (0.0%)	0.458
Hyperthyroidism	1 (3.7%)	1 (3.1%)	>0.999
Kidney failure	0 (0.0%)	0 (0.0%)	-
Asma/DPOC	1 (3.7%)	2 (6.3%)	>0.999
Depression	1 (3.7%)	1 (3.1%)	>0.999
Rheumatoid arthritis	2 (7.4%)	2 (6.3%)	>0.999
Fibromyalgia	2 (7.4%)	5 (15.6%)	0.437
Others	3 (11.2%)	8 (25.0%)	0.172
**Levels with osteoarthritis**			**0.190**
1	1 (3.7%)	6 (19.4%)	
2	9 (33.3%)	4 (12.9%)	
3	9 (33.3%)	13 (41.9%)	
4	5 (18.5%)	6 (19.4%)	
5	3 (11.2%)	2 (6.4%)	

### Oswestry

Table 2 shows the descriptive measures of the Oswestry Disability Index (ODI) according to treatment and follow-up time. It can be observed that there was a significant interaction effect between treatment and time (p = 0.038), meaning that the two treatments did not exhibit the same behavior of the ODI score over time on average.

**Table 2:** Mean ± standard deviation of the ODI score according to treatment and time.

Treatment	Time			p-value
	Baseline	1 month	3 months	
PRP	21.2 ± 7.3	22.0 ± 8.0	17.4 ± 9.7	0.016
Corticosteroid	21.0 ± 8.3	18.7 ± 8.0	20.0 ± 1.6	0.256
p-value	0.933	0.102	0.299	

**Figure 2:**
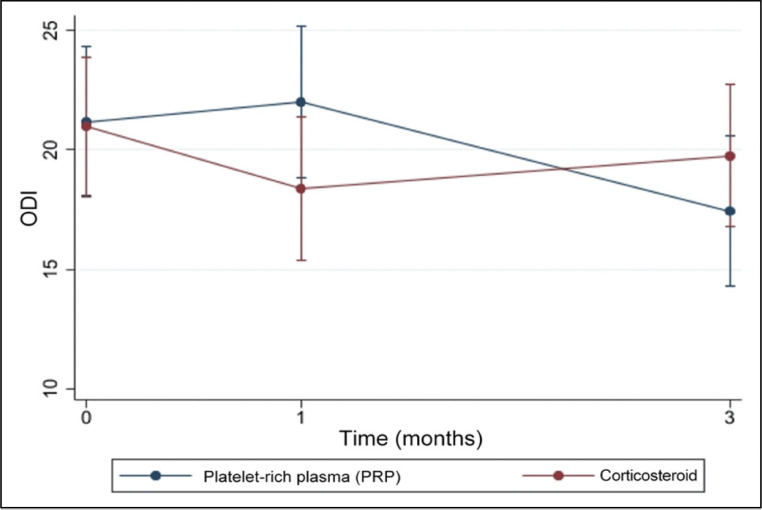
ODI Score. Mean profile (95% CI) of the ODI score over time according to treatment.

This behavior can be seen in Figure 2, where it is noted that from the first to the third month, there is a reversal in the behavior of the average ODI score (p = 0.011), in which in the first month the average observed in the PRP treatment was higher than that of the corticosteroid treatment, but in the third month the average observed in the PRP treatment was lower than that of the corticosteroid treatment.

On the other hand, it was found that there was no significant difference in the average ODI score between the two treatments at any of the evaluation moments (p > 0.05).

### Visual Analog Scale

**Figure 3:**
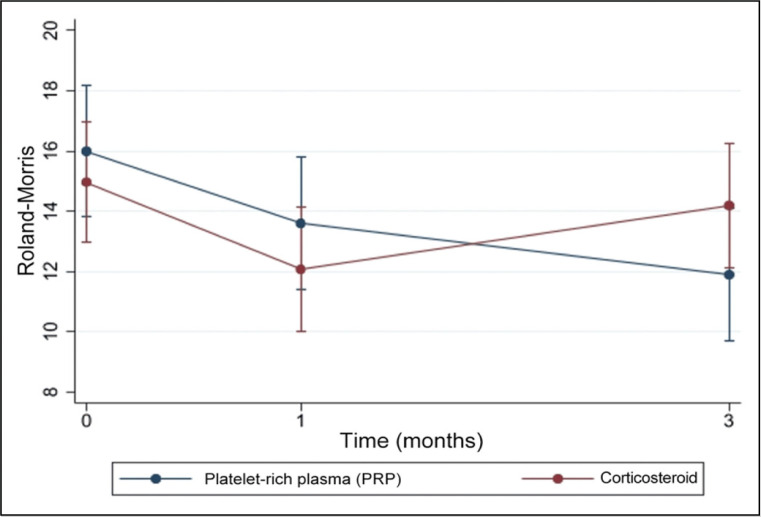
Visual Analog Scale for Pain. Mean profile (95% CI) of VAS over time according to treatment.

Table 3 shows the descriptive measures of the Visual Analog Scale (VAS) for pain according to treatment and follow-up time. It can be observed that there was no significant interaction effect between treatment and time (p = 0.119), meaning that the two treatments exhibited the same behavior of VAS over time on average. Furthermore, it was found that there was no significant difference between the two treatments over time (p = 0.689). Figure 3 illustrates the average profile of the treatments throughout the evaluation.

**Table 3:** Mean ± standard deviation of the visual analog scale (VAS) of pain according to treatment and time.

Treatment	Time			p-value
	Baseline	1 month	3 months	
PRP	71.0 ± 23.1	59.9 ± 25.3	52.1 ± 24.8	0.012
Corticosteroid	76.9 ± 23.2	51.1 ± 27.5	60.4 ± 29.4	<0.001
p-value	0.391	0.210	0.225	

### Roland-Morris

**Figure 4:**
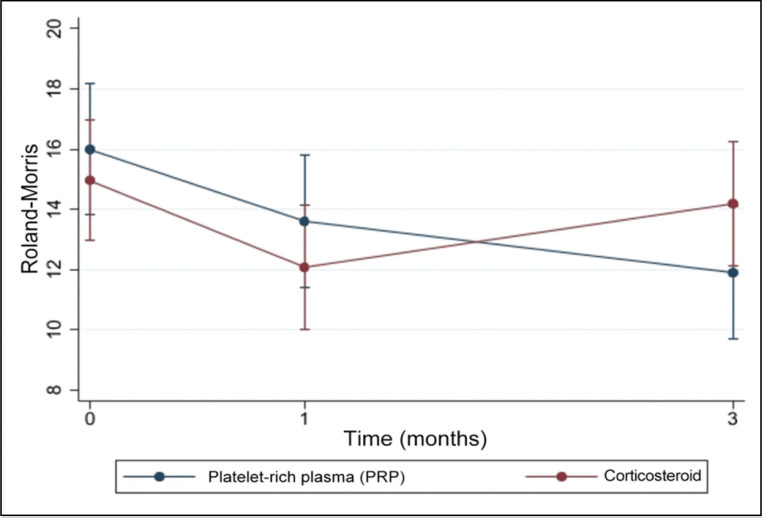
Roland-Morris score. Mean profile (95% CI) of the Roland-Morris score over time according to treatment.

Table 4 shows the descriptive measures of the Roland-Morris score according to treatment and follow-up time. It can be observed that there was a significant interaction effect between treatment and time (p = 0.010), meaning that the two treatments did not exhibit the same behavior of the Roland-Morris score over time on average. This can be seen in Figure 4, where it is noted that from the first to the third month, there is a reversal in the behavior of the average Roland-Morris score (p = 0.010), meaning that in the first month the average observed of the Roland-Morris score in the PRP treatment was higher than that of the corticosteroid treatment, but in the third month the average observed in the PRP treatment was lower than that of the corticosteroid treatment.

**Table 4:** Mean ± standard deviation of the Roland-Morris score according to treatment and time.

Treatment	Time			p-value
	Baseline	1 month	3 months	
PRP	16.0 ± 6.0	13.6 ± 5.8	11.9 ± 5.4	<0.001
Corticosteroid	15.0 ± 4.5	12.3 ± 5.6	14.4 ± 7.2	0.013
p-value	0.498	0.321	0.133	

However, it was found that there was no significant difference in the average Roland-Morris score between the two treatments at any of the evaluation moments (p > 0.05).

## DISCUSSION

The literature shows that facet joint disorders are responsible for 15% to 50% of cases of chronic low back pain^13,14^. In our study, of the 92 patients with chronic low back pain who underwent test infiltration of the medial branch of the facet joint, 60 (65%) patients reported pain relief (VAS ≥ 50%), suggesting pain of facet origin. In line with the literature, the study showed a prevalence of female sex in both studied groups, PRP and corticosteroid (63% and 78.1%, respectively), as well as an average age over 50 years^15^. It is worth noting the clinical homogeneity of the patients in all evaluated epidemiological aspects across the two groups, highlighting that the randomization was effective.

In the functional assessment, both the Oswestry score and the Roland-Morris score showed similar patterns in the present study. Although they did not show statistical significance in superiority of PRP compared to corticosteroids, both showed interaction between treatment and time; that is, PRP and corticosteroids exhibited different behaviors over time in the evaluation of these scores. It is important to remember that in these functional scores, the higher the score, the more debilitated the patient is.

When analyzing the graphs of the functional variables (ODI and RM) (Graphs 1 and 3), we noted that a factor that may have been crucial in the lack of observed superiority of the PRP group over the corticosteroid group was the absence of longer follow-up. Given that in both graphs, the trend was for the curves between the PRP group and the corticosteroid group to diverge, we can assume that if we had evaluated the patients at 6 months, some statistical difference might have been evidenced.

In a randomized clinical trial with 46 patients conducted by Wu et al.^16^, which also compared PRP and corticosteroids in patients with facet joint pain, the same trend in ODI and RM functional scores was observed, but with statistical significance.

When we evaluated the pain scale (VAS), this trial did not show a significant difference between PRP and corticosteroids at any of the evaluated time points, nor did it present interaction between time and treatment. This finding does not confirm what other trials have already evidenced regarding the superiority of PRP over corticosteroids in pain relief, especially at 3 and 6 months^16,17^.

Due to the scarcity of literature on PRP in facet syndrome, we greatly benefit from studies on osteoarthritis of peripheral joints, among which the knee is the target of the largest number of publications on PRP in joints^18^. In the review by Dong et al.^18^, a benefit was evidenced in the use of PRP in knee osteoarthritis, mainly in improving pain and function in the short and medium term (1, 2, 3, 6, and 12 months). The same was not evidenced for the use of PRP in hip osteoarthritis, where the findings were still conflicting regarding pain and function improvement, which was also observed in our study^18^.

On the other hand, in a large recent randomized clinical trial not included in Dong's systematic review et al.^18^, PRP was compared with placebo in knee osteoarthritis^19^ and contrary to the findings of the systematic review, there was no difference at 12 months for pain outcomes and cartilage characteristics between the two groups. In this aspect, the lack of effect of PRP on pain assessment was also found in our trial.

This effect of non-superiority of PRP for pain improvement may reside in the fact that in both cases, the chosen times for follow-up may not have been adequate. It is known that the effect of PRP generally has its best effect between 3 and 12 months^3,20^, and both in the present trial, where patients were evaluated only for 3 months, and in the clinical trial comparing PRP and placebo in knee osteoarthritis, where patients were followed up only at 2 and 12 months, this optimal window was not respected.

Evaluating the limitations that this study presents, we can first cite the sample size. However, even with a reduced sample, this is the largest series in the literature studying PRP in lumbar facet syndrome. Another limitation in the present study is related to the short follow-up. Considering these points, it is of fundamental importance that new randomized controlled clinical trials, with a homogeneous population, reproducible methodology, longer follow-up, and especially larger sample sizes be conducted, so that we can draw better conclusions about the two treatment methods in lumbar facet syndrome.

## CONCLUSION

The present study showed results that evidence the absence of superiority of PRP compared to corticosteroids in pain assessment and functional scores in the treatment of lumbar facet syndrome up to 3 months, making it impossible to see a difference between the methods in this sample.

## Data Availability

The underlying content of the research text is not contained in the manuscript and will be available upon request from reviewers.
